# Predictability of tooth rotations in patients treated with clear aligners

**DOI:** 10.1038/s41598-024-61594-2

**Published:** 2024-05-18

**Authors:** Vincenzo D’Antò, Roberto Rongo, Sossio Dario Casaburo, Stefano Martina, Paolo Petrucci, Kreshnik Keraj, Rosa Valletta

**Affiliations:** 1 Department of Neurosciences, Reproductive Sciences and Oral Sciences, School of Orthodontics, University of Naples Federico II, Via Pansini, 5, 80131 Naples, Italy; 2 Department of Medicine, Surgery and Dentistry “Scuola Medica Salernitana”, University of Salerno, Via Al-Lende, 84081 Baronissi, Italy; 3 Department of Prosthodontic, Faculty of Dental Medicine, University of Medicine, Rruga e ”Dibrës”, AL1005 Tirana, Albania

**Keywords:** Clear aligners, Rotation, Predictability, Accuracy, Orthodontics, Malocclusion

## Abstract

Clear aligners are employed daily for the treatment of several malocclusions. Previous clinical studies indicated low accuracy for the correction of tooth rotations. The aim of this study was to evaluate the predictability of tooth rotations with clear aligners. The sample comprised 390 teeth (190 mandibular; 200 maxillary), measured from the virtual models of 45 participants (21 men, 24 women; mean age: 29.2 ± 6.6 years old). For each patient, pre-treatment (T0) digital dental models (STL files), virtual plan (T1) and post-treatment digital dental models (T2) of both the mandibular and maxillary arches were imported onto Geomagic Control X, a 3D metrology software which allows angular measurements. Rotations were calculated by defining reproducible vectors for all teeth in each STL file and superimposing both T0 with T1 to determine the prescribed rotation, and T0 with T2 to determine the achieved rotation. Prescribed and achieved rotations were compared to assess movement’s accuracy. The Wilcoxon signed-rank test and paired t-test were used to assess differences between the prescribed and achieved movements (*P* < 0.05). The overall predictability of rotational movement was 78.6% for the mandibular arch and 75.0% for the maxillary arch. Second molar accuracy was the lowest in both arches. Clear aligners were not able to achieve 100% of the planned movements.

## Introduction

Clear aligner therapy has become quite popular and nowadays are used to treat various kinds of malocclusion such as Class II, Class III and interdisciplinary cases^1–6^ also considering some of their advantages such as greater ease to maintain good gingival health, lower impact on general well-being and less visibility^7,8^. However, despite the wide use of aligners, they still present an extreme variability in the movement accuracy^9^. In fact, during the treatment with clear aligners many clinicians noticed a difference between the planned movement and the achieved movement^10^ and they had to request at least a case refinement or to pass to fixed appliance to finalize the orthodontic therapy^11,12^. The effectiveness of clear aligners depends mainly on the type of movement required, with overall dental movement predictability varying between 55 and 72%^13^. As already reported in the literature, rotational movement is difficult to achieve with clear aligners; in particular, round-shaped teeth such as canines and premolars often present the lowest accuracy. A systematic review showed that rotational movements have the highest level of inaccuracy when considering prescribed and achieved tooth displacement^14^. A prospective study conducted on a sample of 53 canines in 21 subjects found a very low average canine rotation accuracy, about 36%, which rises slightly (43%) if interproximal reduction (IPR) was performed concurrently^15^. A second study^16^ reported that among mandibular teeth rotation, canine rotation is the least precise (54.2%), while molars show the highest accuracy (85.4%). In the same study, among the teeth of the upper arch, premolars showed the lowest precision (54%) and molars the highest (78%). In a prospective study, Kravitz et al.^17^ reported that only rotation had a significant difference in accuracy between teeth, indeed canines showed a lower accuracy respect to incisors in both arches. Finally, Elkholy et al.^18^ investigated the use of attachments for the rotation of mandibular canines: an increased rotational moment seems to be induced, at least in vitro, with vertical-ellipsoid and quarter-sphere attachment geometry. However, the effectiveness of tooth movement is not only related to the virtual treatment plan but also to the physical and chemical characteristics of the materials and the presence of attachments and their shapes^19–22^. Even if nowadays, there are several are brands of aligners that differ in terms of materials, manufacturing procedures and precision of STL models^23–25^, there is scarce literature that evaluated accuracy of tooth movement with clear aligners different from Invisalign. Therefore, considering the information present in the literature, the purpose of this study was to evaluate the efficacy of rotational movement of maxillary and mandibular teeth with Ordoline clear aligners.

## Methods

The research protocol was designed in accordance with The Code of Ethics of the World Medical Association (Declaration of Helsinki) for experiments involving humans and was reviewed and approved by the Research Ethics Board of the University of Naples Federico II Prot. 352/21. Informed consent was obtained from all the participants.

### Inclusion/exclusion criteria

The sample comprised 390 teeth (190 mandibular; 200 maxillary), measured from the virtual models of 45 participants (21 men, 24 women; mean age: 29.2 ± 6.6 years old). All patients were adults, and only non-extractive treatments were included in the study, except for third molar extraction. Therapy was based only on aligner treatment, thus no combined therapies with fixed appliances or TADs were performed. Patients with general systemic conditions or craniofacial syndromes were excluded from the study. Prescribed rotations of less than 2° were systematically excluded from the study.

### Treatment protocol

Ordoline aligners (UAB Ordoline, Vilnius, Lithuania) were employed in this study. Tooth attachments varied in shape, size, and position according to the tooth typology. In details, for first and second molars rectangular horizontal attachments were used, for central incisors, canines, first and second premolars rectangular vertical attachment were used, for lateral incisors Sash attachment were used. No auxiliaries of any kind were used (intermaxillary elastics, buttons, power chains), although stripping was allowed. Patients were instructed to wear each aligner 22 h per day (except for mealtimes and oral hygiene procedures), 7 days a week; patients were asked to change aligners every 10 days.

### Data collection

Using an intraoral scanner (IOS), two digital impressions of the mandibular and maxillary arches were recorded for all patients. The first digital impression was named T0 (STL file from before the start of treatment); the second impression was named T2 (STL at the end of the first set of aligners). T1, on the other hand, corresponds to the final virtual setup. The virtual plan STL file was exported from the dedicated planning software to compare with the other digital impressions.

### Measurement methodology

**Figure 1:**
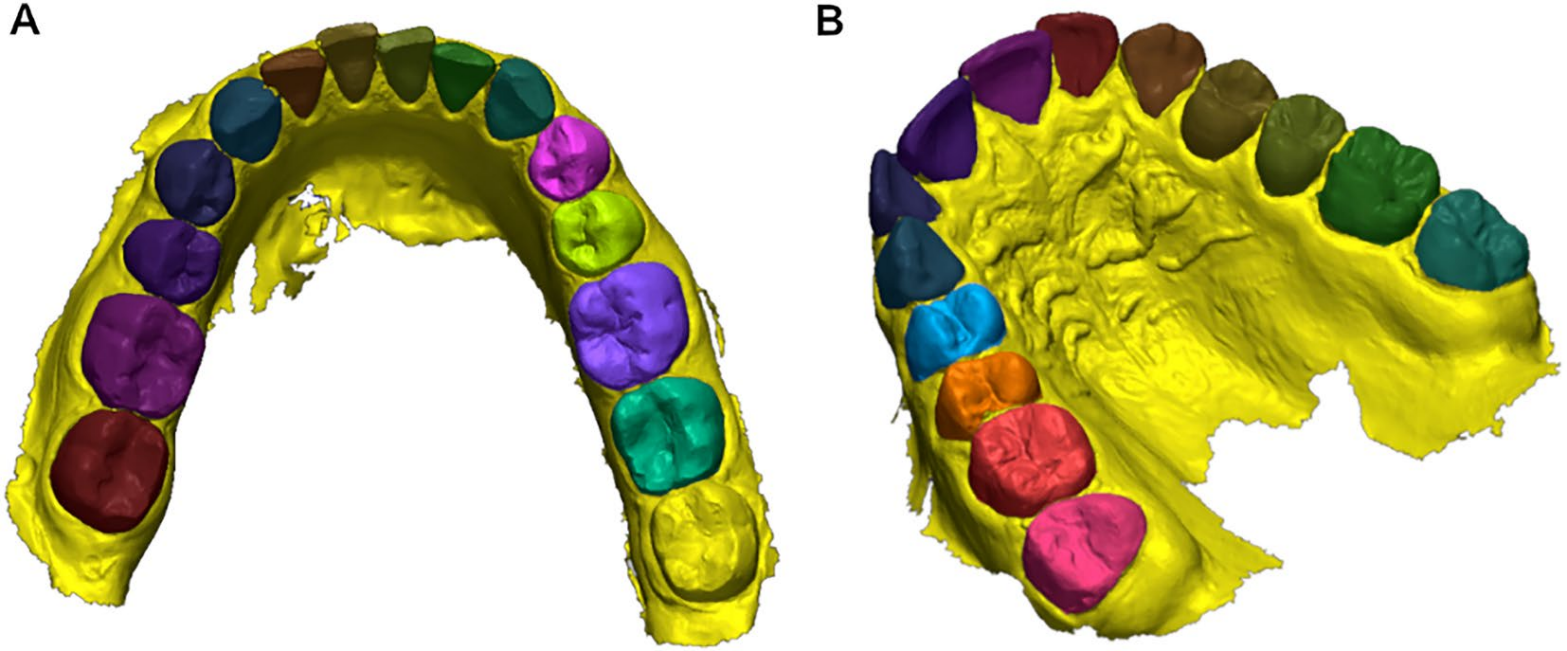
Teeth segmentation. (**a**) Mandibular teeth segmentation. (**b**) Maxillary teeth segmentation.

STL files were uploaded to Geomagic Control X 2021.0.0 (3D Systems, Rock Hill, SC, USA), a professional 3D dimensional inspection and quality control software used to acquire and process data from 3D scanners. T0 and T1 were initially compared and superimposed to establish the amount of rotation prescribed. T0 was assigned to “reference data” and T1 to “measured data”, then each tooth was segmented in T0 (Fig. 1). Two landmarks for each tooth were selected to define a vector:

- Disto-vestibular and mesio-lingual cusps for molars;
- Vestibular and lingual cusps for premolars;
- The most mesial and most distal points of the incisal edge for incisors and canines.

**Figure 2:**
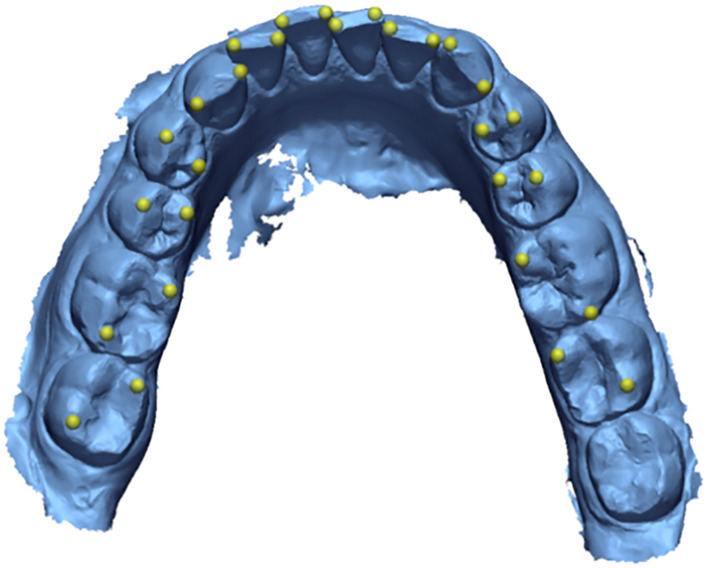
Example of mandibular landmarks positioning.

**Figure 3:**
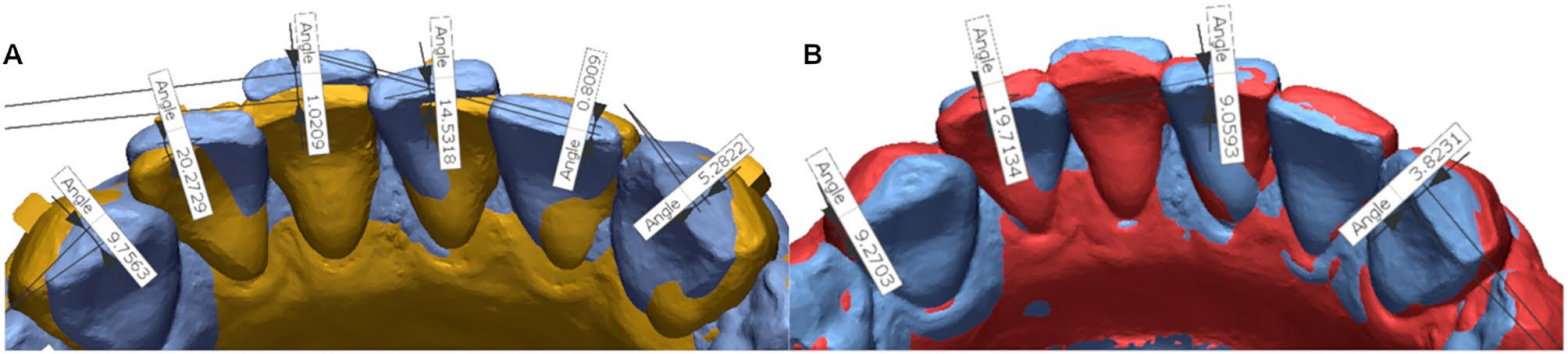
Example of digital model superimposition and rotational movement calculation. (**a**) Superimposition of T0 (blue) with T1 (yellow); prescribed angular variation. (**b**) Superimposition of T0 (blue) with T2 (red); achieved angular variation.

All desired landmarks were placed by an operator in T0 (Fig. 2). Then, with the purpose of maintain the same anatomical location of the landmarks also in T1, each tooth previously segmented was sequentially chosen as reference for a surface-based best fit between T0 and T1. The new landmarks on T1 were created in the same position of the landmarks in T0. By subsequently changing the alignment of the STL files in search of the global best-fit, the landmarks automatically adapted to the displacement. For each tooth, the same procedure was repeated. Regarding global alignment, the methodology implemented is like the one described by Grunheid et al.^26^. After an initial alignment based on three points (the tip of the mesio-buccal cusp of the right and left first molars and the most mesial point of the incisal edge of the right incisor), the superimposition was finalized using the global best-fit function set to 50 iterations as suggested by the most recent literature^26,27^. The same three points were consequently adopted to delineate the occlusal plane on T0. To calculate the prescribed angular variation, a vector passing through the two landmarks was traced for each tooth on both T0 and T1. All vectors were then projected onto the occlusal reference plane and the angle between the two vectors was calculated. The T2 STL file was uploaded to Geomagic instead of T1 and compared with T0, with the purpose of analyze the rotation achieved, and the previous procedures was used also to analyze T2 respect to T0 (Fig. 3).

### Outcome assessed

Prescription, achieved movement, accuracy and performance for each tooth rotation was analyzed according to the following variables:

- Prescription represents the amount of rotation resulting from the superposition between the pre-treatment (T0) and the virtual plan (T1).
- Achieved movement represents the amount of rotation resulting from the superposition between pre-treatment (T0) and post-treatment (T2).
- Accuracy was calculated as follows:
  $$ {\text{Accuracy}}\left( \% \right) = \left( {{\text{Achieved}}/{\text{Prescription}}} \right)*{1}00. $$
- Mean performance was determined in absolute value as the difference between the achieved and prescribed rotation to emphasize the degree of accuracy of the rotation rather than the direction for all the teeth.

Precise rotation is defined as rotation whose performance is close to 0°; the further the performance is from the ideal value of 0°, the more inaccurate the rotation. The frequencies of performance error index (FOPE) was then divided into four ranges: less than or equal to 1°, between 1.1° and 2°, between 2.1° and 4°, and greater than 4° considering the absolute values. Then to assess the direction of the inaccuracy, three variables were evaluated:

- Under-performance (differences between achieved and prescribed rotation less than − 1°);
- Right-performance (differences between achieved and prescribed rotation between − 1° and 1°);
- Over-performance (differences between achieved and prescribed rotation greater than 1°).

### Statistical analysis

Considering that the premolars are usually the teeth that have the worst performance for rotation, we set the discrepancy between achieved movement and prescribed movement for the premolar rotation as the main outcome. When considering a *P* value of 0.05 and an effect size of 0.6^17^, a sample size of 24 teeth is sufficient to achieve a power of 80%, using a t-test as statistical analysis. The ICC index, the Dahlberg error assessment, and the T test for paired data (*P* < 0.05) were used to evaluate the intra-operator and inter-operator error of the method. To assess the reproducibility of the method, 30% of the rotations were recalculated by both the same operator and another operator at 5 weeks apart. The statistical package SPSS (IBM, Chicago, IL, USA) was used. Descriptive statistical analysis included means, standard deviations, and C.I. 95% for prescription, achieved movement and accuracy. A Shapiro–Wilk normality test was performed to assess the distribution of the data. A Wilcoxon signed-rank test and paired t-test were used to evaluate whether the difference between prescription and achieved movement was statistically significant. The significance level was set at 0.05. A regression analysis was performed to assess the influence of the amount of prescription on the total accuracy. The significance level was set at 0.05. Considering the inherent superposition error of the STL files and the low statistical significance, a clinical significance cut-off of 2° was set. Prescribed rotations of less than 2° were systematically excluded from the study.

### Ethics approval and consent to participate

The study protocol complied fully with the principles of the Helsinki Declaration and was approved by the Ethics Committee of the University Federico II (352/21).

## Results

### Intra-operator and inter-operator error of the method

The ICC for intra-operator and inter-operator reproducibility was respectively 0.988 and 0.996 for Prescription, 0.989 and 0.986 for Achieved movement. The technical error of the method of the Prescription was respectively 0.89 and 0.55, and of the Achieved movement was 0.53 and 0.63. The paired T test did not show any statistically significant differences for Prescription and Accuracy for both intra-operator and inter-operator evaluation (*P* > 0.05).

### Prescription versus achieved movement

Table 1 shows mean, standard deviation, the upper limit and lower limit of 95% confidence intervals for Prescription, Achieved movement and Mean performance. Statistically significant differences were found for all the teeth except for the mandibular central incisor (*P* > 0.05). Regarding the lower arch, the lowest rotational rate was prescribed to the first molar, which also reported the lowest rotation achieved; in the upper arch, instead, the first molar reported both the highest Prescription and Achieved movement (Table 1).

**Table 1:** Descriptive statistics of prescription and achieved movement.

		Prescription (°)		Achieved movement (°)		Mean performance (°)		*P* vs AM
	n	Mean ± SD	95% CI LL–UL	Mean ± SD	95% CI LL–UL	Mean ± SD	95% CI LL–UL	*P-*value
Mandibular central incisor	30	8.79 ± 5.84	6.61–10.97	7.36 ± 6.06	5.10–9.63	1.42 ± 4.07	− 0.10–2.94	0.086*
Mandibular lateral incisor	30	10.77 ± 9.50	7.21–14.31	8.84 ± 7.97	5.86–11.81	1.93 ± 5.34	− 0.06–3.93	0.045*
Mandibular canine	30	11.03 ± 6.94	8.44–13.62	8.46 ± 5.81	6.29–10.64	2.57 ± 3.46	1.28–3.86	** < 0.001**
Mandibular first premolar	30	11.48 ± 7.02	8.86–14.10	8.48 ± 7.05	5.84–11.11	3.01 ± 3.08	1.86–4.16	< 0.001*
Mandibular second premolar	30	10.18 ± 11.25	5.98–14.38	4.17 ± 7.34	4.17–9.66	3.27 ± 7.66	0.41–6.13	0.001*
Mandibular first molar	20	5.55 ± 2.99	4.14–6.95	3.88 ± 2.57	2.68–5.08	1.67 ± 2.83	0.34–2.99	**0.016**
Mandibular second molar	20	6.12 ± 3.58	4.45–7.80	4.15 ± 2.75	2.86–5.44	1.98 ± 2.53	0.80–3.16	0.002*
Total mandibular	190	9.48 ± 7.74	8.37–10.59	7.17 ± 6.25	6.25–8.09	2.31 ± 4.59	1.65–2.97	< 0.001*
Maxillary central incisor	30	7.52 ± 4.05	6.00–9.03	5.51 ± 3.30	4.28–6.74	2.01 ± 2.20	1.19–2.83	< 0.001*
Maxillary lateral incisor	30	7.62 ± 4.84	5.81–9.43	5.56 ± 4.62	3.83–7.29	2.07 ± 2.84	1.00–3.13	0.001*
Maxillary canine	30	8.32 ± 6.21	5.99–10.64	5.41 ± 4.35	3.79–7.04	2.91 ± 4.79	1.12–4.70	0.002*
Maxillary first premolar	30	6.92 ± 3.56	5.59–8.26	5.46 ± 3.29	4.23–6.69	1.47 ± 2.41	0.57–2.37	**0.002**
Maxillary second premolar	30	7.85 ± 5.84	5.67–10.04	6.60 ± 5.56	4.53–8.68	1.26 ± 2.61	0.28–2.23	0.022*
Maxillary first molar	25	10.35 ± 5.50	8.07–12.62	7.90 ± 4.80	5.92–9.88	2.45 ± 2.90	1.26–3.65	** < 0.001**
Maxillary second molar	25	10.14 ± 6.88	7.29–12.98	5.37 ± 3.49	3.94–6.81	4.77 ± 5.13	2.65–6.88	** < 0.001**
Total maxillary	200	8.30 ± 5.39	7.55–9.05	5.94 ± 4.30	5.34–6.54	2.36 ± 3.52	1.87–2.85	< 0.001*

*Wilcoxon signed-rank test. °, degrees; CI 95% LL, confidence interval 95% lower limit; CI 95% UL, confidence interval 95% upper limit; Mean performance, absolute value of the mean difference between Achieved movement and Prescription. Bold text indicates statistically significant differences.

### Accuracy

Table 2 shows mean, standard deviation, the upper limit and lower limit of 95% confidence intervals for accuracy. About the lower arch, the highest accuracy was reported for the rotation of the lateral incisor (89.6%), the lowest accuracy was reported for the rotation of the second molar (70.9%), and the overall accuracy was 78.6%. For the upper arch, the highest accuracy was reported for the rotation of the second premolar (82.0%), the lowest accuracy was reported for the rotation of the second molar (60.7%), and the overall accuracy was 75.0%.

**Table 2:** Descriptive statistics of accuracy.

	Accuracy		
	n	Mean ± SD	95% CI LL–UL
Mandibular central incisor	30	88.34 ± 49.83	69.73–106.95
Mandibular lateral incisor	30	89.61 ± 43.83	73.25–105.98
Mandibular canine	30	78.13 ± 33.26	65.71–90.55
Mandibular first premolar	30	71.14 ± 30.62	59.70–82.57
Mandibular second premolar	30	73.39 ± 37.43	59.41–87.36
Mandibular first molar	20	74.68 ± 45.76	53.27–96.10
Mandibular second molar	20	70.94 ± 29.74	57.02–84.86
Total mandibular	190	78.58 ± 39.49	72.93–84.23
Maxillary central incisor	30	75.55 ± 27.29	65.36–85.74
Maxillary lateral incisor	30	73.90 ± 33.90	61.25–86.56
Maxillary canine	30	73.59 ± 45.95	56.44–90.75
Maxillary first premolar	30	79.21 ± 31.90	67.30–91.12
Maxillary second premolar	30	82.03 ± 38.46	67.67–96.39
Maxillary first molar	25	78.39 ± 29.52	66.21–90.58
Maxillary second molar	25	60.70 ± 31.65	47.64–73.76
Total maxillary	200	75.03 ± 34.80	70.18–79.88

CI 95% LL, confidence interval 95% lower limit; CI 95% UL, confidence interval 95% upper limit.

### FOPE

Table 3 shows the FOPE calculated as absolute values for each type of tooth and the distribution of under-performance, right-performance, and over-performance. For the lower arch, the best FOPE was detected for the second premolar (16 times, 53.3%). For the upper arch, the best FOPE was detected for the first premolar (12 times, 40%). About the direction of the inaccuracy, for the lower arch, the second premolar showed the highest frequency of right-performance (53.3%), the canine showed the highest frequency of under-performance (63.3%), while the central incisor showed the highest frequency of over-performance (26.7%). The overall right-performance was 32.1%, the overall under-performance was 55.3%, and the overall over-performance was 12.6%. Regarding the upper arch, the first premolar showed the highest frequency of right-performance (40%), the second molar showed the highest frequency of under-performance (76%), and the canine and second premolar showed the highest frequency of over-performance (16.7%). For the whole sample, the total right-performance was 30.5%, the total under-performance was 58.5%, and the total over-performance was 11%.

**Table 3:** FOPE of the achieved movements.

	FOPE							
	n	Under-performance	Right-performance	Over-performance	Performance ≤ 1 *n %*	1 < Performance ≤ 2 *n %*	2 < Performance ≤ 4* n %*	Performance > 4 *n %*
Mandibular Central Incisor	30	16 (53.3%)	6 (20%)	8 (26.7%)	6 (20%)	4 (13.3%)	10 (33.3%)	10 (33.3%)
Mandibular Lateral Incisor	30	15 (50%)	9 (30%)	6 (20%)	9 (30%)	7 (23.3%)	8 (26.7%)	6 (20%)
Mandibular Canine	30	19 (63.3%)	8 (26.7%)	3 (10%)	8 (26.7%)	5 (16.7%)	9 (30%)	8 (26.7%)
Mandibular First Premolar	30	18 (60%)	11 (36.7%)	1 (3.3%)	11 (36.7%)	3 (10%)	4 (13.3%)	12 (40%)
Mandibular Second Premolar	30	13 (43.3%)	16 (53.3%)	1 (3.3%)	16 (53.3%)	4 (13.3%)	4 (13.3%)	6 (20%)
Mandibular First Molar	20	13 (65%)	3 (15%)	4 (20%)	3 (15%)	8 (40%)	4 (20%)	5 (25%)
Mandibular Second Molar	20	11 (55%)	8 (40%)	1 (5%)	8 (40%)	3 (15%)	5 (25%)	4 (20%)
Total Mandibular	190	105 (55.26%)	61 (32.11%)	24 (12.63%)	61 (32.11%)	34 (17.89%)	44 (23.16%)	51 (26.84%)
Maxillary Central Incisor	30	19 (63.3)	9 (30%)	2 (6.7%)	9 (30%)	10 (33.3%)	5 (16.7%)	6 (20%)
Maxillary Lateral Incisor	30	19 (63.3)	8 (26.7%)	3 (10%)	8 (26.7%)	12 (40%)	2 (6.7%)	8 (26.7%)
Maxillary Canine	30	15 (50%)	10 (33.3%)	5 (16.7%)	10 (33.3%)	4 (13.3%)	4 (13.3%)	12 (40%)
Maxillary First Premolar	30	16 (53.3%)	12 (40%)	2 (6.7%)	12 (40%)	9 (30%)	5 (16.7%)	4 (13.3%)
Maxillary Second Premolar	30	15 (50%)	10 (33.3%)	5 (16.7%)	10 (33.3%)	9 (30%)	6 (20%)	5 (16.7%)
Maxillary First Molar	25	14 (56%)	8 (32%)	3 (12%)	8 (32%)	4 (16%)	4 (16%)	9 (36%)
Maxillary Second Molar	25	19 (76%)	4 (16%)	2 (8%)	4 (16%)	9 (36%)	3 (12%)	9 (36%)
Total Maxillary	200	117 (58.5%)	61 (30.5%)	22 (11%)	61 (30.5%)	57 (28.5%)	29 (14.5%)	53 (26.5)

FOPE, Frequencies of performance error; Under-performance, performance < − 1°; Right-performance, − 1° ≤ performance ≤ 1°; Over-performance, performance > 1°

### Regression analysis

Table 4 shows the regression analysis assessing the influence of the amount of prescription on the total accuracy. No statistically significant differences were found for any tooth typology.

**Table 4:** β, changes in accuracy when increasing a single degree of prescription.

	Prescription increase		
	β	95% CI LL–UL	*P-*value
Mandibular central incisor	− 1.21	− 4.48–2.06	0.455
Mandibular lateral incisor	− 0.932	− 2.68–0.82	0.284
Mandibular canine	− 0.339	− 2.19–1.51	0.710
Mandibular first premolar	0.643	− 1.03–2.31	0.437
Mandibular second premolar	− 0.456	− 1.73–0.82	0.470
Mandibular first molar	− 3.100	− 10.51–4.31	0.391
Mandibular second molar	− 1.635	− 5.67–2.40	0.406
Maxillary central incisor	− 1.075	− 3.646–1.495	0.399
Maxillary lateral incisor	− 0.336	− 3.039–2.367	0.801
Maxillary canine	− 1.901	− 4.664–0.863	0.170
Maxillary first premolar	− 0.228	− 3.69–3.23	0.894
Maxillary second premolar	0.474	− 2.07–3.02	0.706
Maxillary first molar	− 0.746	− 3.03–1.54	0.507
Maxillary second molar	− 1.716	− 3.56–0.12	0.066

CI 95% LL, confidence interval 95% lower limit; CI 95% UL, confidence interval 95% upper limit.

## Discussion

The aim of this study was to assess the predictability of the rotational movement of both maxillary and mandibular teeth in patients treated with clear aligners by evaluating the discrepancies between the virtually planned (T0–T1 superimposition) and the clinically achieved (T0–T2 superimposition) angle of rotation. Except for the lower central incisor, statistically significant differences were found for all the other teeth, meaning that the rotation achieved with aligners during treatment was not accurate. However, accuracy did not explain the amount and the direction of this discrepancy; therefore, FOPE was introduced to analyze both the severity of the inaccuracy and the quality of the performance. Indeed, the accuracy value alone (achieved movement/prescribed) is not sufficient to properly assess how well the aligners perform regarding rotational movement. FOPE can give more appropriate clinical information on what we could expect in terms of amount of discrepancy, while over and under performance during rotational movements, indicate the direction of the inaccuracy. The FOPE was calculated in absolute value (|achieved movement—prescription|divided into ranges) to assess the number of discrepancies independently by the direction, and the direction of inaccuracy was calculated in relative value to evaluate the amount of right, over and under performance. In general, orthodontic movement depends on the anatomy of the crown and roots of the teeth^26^ so the results were evaluated divided by tooth typologies.

### Incisors

An accuracy of 88.3% and of 75.5% were found respectively for the mandibular and maxillary central incisor but considering the low frequencies of right performance and the high value of FOPE > 4, the high accuracy value was not due to a high frequence of right-performance but rather was consequence of the number of over-performances that produce accuracies higher than 100%, that increase the mean accuracy of the movement. The rotations of upper and lower central incisors present a good accuracy, with lower arch achieving better results than upper arch^11,17,28,29^, but the possibility of an excessive correction of the rotation should be taken into account. The mandibular lateral incisor showed a mean accuracy of 89.6%, the highest of the entire study in accordance with a previous work^30^. Like the central mandibular incisor, an over-performance was observed in 1 case out of 5 (20%). Considering a lower frequency of 2 < FOPE < 4 and FOPE > 4, the accuracy of the lateral incisor is more consistent than the central incisor. There also seems to be a trend of better accuracy for the lower lateral incisor than for the upper lateral incisor. It is interesting to note the divergence in behavior of the incisors between the mandibular and maxillary arches. At the lower arch, the lateral incisor showed higher accuracy than the central incisor, which is the opposite for the upper arch.

### Canines and premolars

In our study we observed a relatively high accuracy value for the mandibular canine associated with high frequency of under-performance (63.3), while the maxillary canine showed the worst behavior after the second molar, confirmed by a FOPE > 4 of 40%. Concerning the lower arch, the premolars were undoubtedly, excluding the second molars, the teeth that had the worst behavior. Only once for each lower premolar we noticed a level of accuracy above 100% (over-performance 3.3%) and the frequencies of under-performance were much larger (60% for the first premolar and 43.3% for the second premolar). In particular, the lower first premolar reported the worst accuracy levels of the entire study, as supported by a FOPE > 4 (40%). Certainly, it was an expected outcome, since canines, first and second premolars are called “round teeth” because of their round-conical shape. Specific consideration should be made for these teeth. According to current literature, round teeth are the ones that hardly achieve the prescribed rotation^14^, probably because clear aligners, as thermoplastic appliances, tend to lose anchorage and slip off due to the limited undercuts and the round tooth shape. It seems that there are methods to improve the effectiveness of the rotational movement of round teeth. Particularly, in 2014 Simon et al.^31^ described that, with the support of an attachment, the accuracy increased from 42.4 to 47.3%, however in our study the accuracies of all teeth in upper and lower arch were higher. This increase in the accuracy might be due to the improvement of aligner materials, and to the use of rectangular vertical attachments that were used in canines and premolars in both upper and lower arch. Indeed, studies evaluated the use of different attachments for the rotation of premolars and canines, in both cases the rectangular vertical attachment was used with good results, even if for lower canines the use of the optimized rotation attachments using 1-week wear was suggested^32^. Finally, IPR (interproximal reduction) increase the accuracy of rotational movement compared to the same movement without IPR, identifying the presence of an interproximal contact as a crucial element in the success of canine rotation^15^.

### Molars

Mean accuracy was 74.7% for the mandibular first and 78.4% for the maxillary first molar. We found a huge difference in accuracy between the first and second molars for both arches. The maxillary second molar reported the lowest accuracy among all teeth and the highest frequency of under-performance in the entire study. This was an expected result since second molars usually have no distal teeth, have a short crown, and are consequently difficult to move with clear aligners. There are few studies that investigated molar rotations. D’Antò et al.^25^ analyzed the rotation with clear aligners by calculating the FOPE. Compared to our study, regarding the maxillary first molar, a similar outcome of FOPE < 1 was found. More recently, a detailed study based on more than 2,000 teeth by Castroflorio et al.^33^ used the "lack of correction index" which indicates loss of prescribed movement, to analyze all types of movement and rotations. According to the authors, first molars and molars in general are teeth that showed the best reproducibility, however this evaluation was based only on the achieved movement and not on the comparison between prescribed and achieved movement, so it was not possible to establish the performance of the movement.

In the present study it was not possible to find a statistically significant association between the increase of the prescribed angle of rotation and the accuracy, for any tooth. This is in contrast with a recent study that found several factors that influence accuracy of tooth rotation such as: patient’s age, tooth type, the magnitude of the predicted rotational movement, and whether or not IPR was prescribed^34^. The main difference between these two studies is that in the study of Xie et al.^34^, were included only anterior teeth that needed more than 15°, while in our study the average rotation prescribed was 7°, it could be hypothesize that for anterior teeth if big rotations are required these can influence the accuracy of the movement.

To quantify the amount of rotation of the teeth included in the sample, it was necessary to superimpose the digital models. All data in this and similar studies can only be considered reliable if the methodology of superimposing STL files is extremely accurate, in this study the superposition methodology already suggested by Grunheid et al.^26^ was followed as described before. This method has recently been used by other authors^29,35^ and shows excellent reproducibility; furthermore, this choice is fully supported by recent research studies, which indicate that the software’s general best-fit system is reliable and very similar to the semi-automatic superimposition based on palatine rugae^27,36^. The inherent risk of repositioning landmarks on the three STL files (T0–T1–T2) was addressed in three different ways. This was a delicate step since a slight mismatch in the repositioning of the landmarks could alter the result. First, teeth were segmented and a protocol comprising best-fit superimposition for each tooth and a point positioning system based on coordinate copying ensured that the point chosen was the same on both digital models. Then the ICC was used to assess the reproducibility of the measurements and showed excellent agreement for both intra-examiner (0.988 for prescription and 0.989 for achieved) and inter-examiner (0.996 for prescription and 0.986 for accuracy) reliability. Last, a cutoff of 2° for angular measurements was introduced to account for model superimposition error; values below the cutoffs were considered clinically irrelevant and they could falsely increase the accuracy since small movements usually perform very well^16,37^.

This study presents some limitations: compliance was not assessed but we included only adult patients that arrived at the end of the first step off aligners without extra-refinement and in the correct amount of time. Regarding the attachment, no standardized protocol was used; all patients wore attachments and had IPR, but these varied in size, and position. Indeed, same teeth had same attachments as listed before, and usually they were placed at the center of the tooth when it was possible. Finally, multiple teeth were used from the same patient, but, in reality, the movement of one tooth is not independent of the movement of adjacent teeth or the ones that are used as anchorage and this could explain also the percentage of under and over-performance found, however this scenario represents the normal clinical scenario in everyday orthodontics. Finally the strengths of this study are the information provided also of other brand of aligners and not only on Invisalign, the amount of teeth assessed with a highly reliable method supported by the literature^24,26,27,29,33,35,36,38^, the introduction of two variables “performance” and FOPE that allow to identify not only the accuracy but also the amount and the direction of the discrepancy between prescription and achieved movement.

## Conclusions

To summarize the outcomes of our survey on the predictability of rotational movement of mandibular and maxillary teeth using clear aligners, the following can be concluded:

- The overall accuracy was 78.6% for the mandibular arch and 75.0% for the maxillary arch.
- Excluding the second molars, obvious differences were found between the two arches. At the lower arch, the incisors were the best performing teeth while round teeth showed difficulty in rotational movement. At the upper arch, by contrast, the premolars were the most accurate teeth while incisors were the teeth with the highest frequency of under-performance.
- None of the teeth showed accuracy near or above 100%, so a check at an intermediate phase and a finishing phase is recommended.

## Data Availability

The data presented in this study are available on request from the corresponding author.

## Abbreviations

STL: Stereolithography
IPR: Interproximal reduction
